# Presence of Leptin in Chronic Periapical Lesions

**Published:** 2010-11-15

**Authors:** Ali Kangarlou Haghighi, Mohammad Davar, Majid Kazem, Omid Dianat

**Affiliations:** 1. Department of Endodontics, Iranian Center for Endodontic Research, Dental School, Shahid Beheshti University of Medical Sciences, Tehran, Iran.; 2. Shiraz University of Medical Sciences, Shiraz, Iran.

**Keywords:** Periapical Diseases, Cytokine, Inflammation, Leptin

## Abstract

**INTRODUCTION:**

Studies have shown the regulatory role of Leptin in bone formation, its expression in adipose tissue as well as increased levels in circulation following the adminstration of inflammatory stimuli such as lipopolysaccharides (LPS). However, there is little data evaluating the role of Leptin in inflammatory periapical lesions. The aim of this study was to evaluate the presence and concentration of Leptin in chronic periapical lesions.

**MATERIALS AND METHODS:**

Chronic periapical lesions with different sizes were collected during periapical surgery of the mandibular molars from twenty patients and cultured for 72 hours. The ELISA method determined the concentration of Leptin in supernatant fluids of explants cultures. Statistical analysis was performed using non-parametric tests (Mann-Whitney U, Chi-Square and Spearman’s Correlation Coefficient).

**RESULTS:**

Leptin was found in all samples with the average concentration of 405.55±102.98 (pg/mL). There was no significant correlation between the concentration of Leptin and BMI, and the diameters of lesions.

**CONCLUSION:**

Leptin can be considered an inflammatory mediator and is likely to have a role during the early phases of dental periapical lesions.

## INTRODUCTION

Various factors and mediators of the immune system may be found in inflamed dental pulps; one important family is the cytokines. Bacteria will cause infection-stimulated inflammatory processes within the dental pulp before affecting the periapical region [1][2][3][4][5][6][7]. Bacterial infection of dental pulps, usually as a sequel to dental caries, can result in chronic periapical lesions. This type of lesion is often an immune reaction to the bacteria and by-products of the root canal system, which can ultimately lead to the destruction of periapical and perifurcal alveolar bone.

The inflammatory lesion is characterized by the presence of immune cells which can produce inflammatory mediators, such as cytokines [8][9][10][11][12][13][14]. Leptin is an adipocytokine that plays a central role in metabolism. Recent studies have shown that Leptin may also be involved in the inflammatory responses such as induction of acute phase protein synthesis and stimulation of macrophages and/or T cells. Adipocytes and epithelial cells of the gastrointestinal tract are major cellular producers of Leptin. Recently, the gingiva was also considered as a cellular source of Leptin. This mediator induces its effects through binding to its specific receptors, present on the different cell types such as hypothalamus cells, T cells, endothelial cells, macrophages, pancreatic cells, adipocytes, osteoblasts and hematopoietic cells. Presence of Leptin receptors on the surface of T cells and macrophages and some structural similarities between Leptin and certain cytokines including IL-1, IL-6 and LIF suggest that Leptin could have an important role in the inflammatory response [15][16][17][18].

Johnson and Serio demonstrated the presence of Leptin both in healthy and inflamed oral gingival tissues [19]. However, there are no studies that evaluate the role of Leptin in the inflammatory periapical lesions. The aim of this study was to evaluate the presence and concentration of Leptin in chronic periapical lesions.

## Materials and Methods

Chronic periapical lesions (chronic apical periodontitis) of different sizes were collected by conducting standard periapical surgery on mandibular molars of twenty patients. The average age of patients was 31.60±7.99 yrs; 40% of subjects were female and 60% male. The average BMI of subjects was 24.92±3.44; 15% of patients were underweight and 15% were overweight. Subjects that were included did not have any systemic disease and had not taken antibiotic, nonsteroidal anti-inflammatory drugs, and/or H1 and H2 antagonists in the past 3 months. Lesions that were collected were cultured for 72 hours.

The radiographic appearances of the lesions indicated that 75% were <5mm in diameter and 25% had diameters ≥5mm.

Following surgical excision, all lesions were divided to two pieces; one was used for pathologic evaluation and the other for tissue culture. Pathologic assessment revealed that all lesions were periapical granulomas.

For tissue culture, samples were immediately placed in culture medium [RPMI-1640 (10g/L) and Fetal Bovine Serum (10%)] which kept on ice after surgical excision. The samples were then rinsed with a solution of 10g/L RPMI 1640, 100UI/mL penicillin and 100μg/mL streptomycin. They were cut into small pieces (1mm^3^) with a surgical scalpel in sterile Petri dishes. Each segment (corresponding to one sample) was scratched with sterile surgical blades (No 15) and then moved to a single well of a 96-well tissue culturing plate and submerged in 300µL of a culture medium containing 10g/lit RPMI 1640, Fetal Bovine Serum, Penicillin-Streptomycin (100 UI/mL penicillin and 100μg/mL streptomycin). The plates were then transferred to an incubator with 5% CO_2_ and kept for 72 hours. Tissue culture medium was replenished at 24, 48, and 72 hours. Following each interval, the supernatant medium was extracted with tuberculin syringes and frozen at -70°C. Histopathologic assessment verified the viability of cells after culturing.

In order to determine the presence and concentration of Leptin in supernatant fluids ELISA method was utilized (Bender medsystems ELISA kit, Austria, Cat. No. BMS2039INST).

Statistical analysis was undertaken using non-parametric tests including Mann-Whitney U, Spearman’s Correlation Coefficient and also Chi-Square test.

## Results 

**Figure 1:**
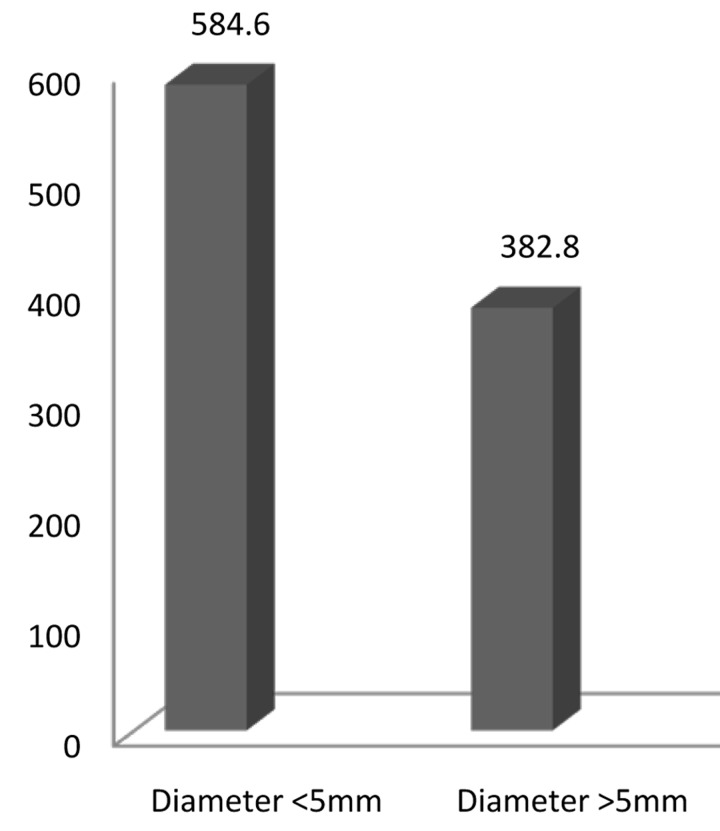
Comparison of Leptin concentration (pg/mL) between the lesions with diameter of <5mm or >5mm

Leptin was discovered in all samples with an average concentration of 405.55±102.98 (pg/mL). There was no significant correlation between the concentration of Leptin and BMI. There was no statistically significant correlation between Leptin concentration and the diameters of lesions; however, the concentration of Leptin was higher in small lesions (<5mm) than large lesions. In Figure 1, the concentration of Leptin in small and large lesions has been compared.

## Discussion

Leptin is a 16 kDa cytokine-like hormone encoded by OB gene and is predominantly expressed in adipose tissues. Due to its high expression in adipose tissues it is known to regulate food intake [15][16]. Some studies report increased levels of Leptin during infectious and inflammatory processes. Leptin expression in adipose tissue and circulating Leptin levels are increased after administration of inflammatory stimuli such as lip polysaccharides (LPS) or turpentine to hamsters [20][21][22]. It was suggested that Leptin acts mostly as a pro-inflammatory factor during adaptive immune responses, whereas in processes involving innate immunity, anti-inflammatory effects of Leptin are prevalent [23]. In addition, Leptin has been shown to regulate bone formation [18]; consequently, there are several studies have assessed the correlation between Leptin and periodontal disease. Johnson and Serio found Leptin both in healthy and inflamed gingival tissues, although its concentration was higher in healthy gingival samples [19]. Karthikeyan and Pradeep reported a negative correlation of GCF Leptin levels with clinical attachment loss and as periodontal tissue destruction increased, there would be a substantial decrease in the concentration of Leptin in GCF [24]. Shimada et al., however, found a decrease in serum Leptin levels following non-surgical periodontal treatment [25], creating controversy about the precise role of Leptin in the pathogenesis of periodontal disease (inflammatory diseases). Leptin is a strong enhancer of adaptive or specific immunity; on the other hand, it can also dampen innate or non-specific immunity. Since the most pathologic changes are mediated by helper T cells, its reparative role remains vague. Further studies are needed to shed light on its role.

The relationship between Leptin and chronic dental periapical lesions has not been previously analyzed. However, neuropeptides have been shown to induce the expression of Leptin in pulpal fibroblasts [26], which indicates that during inflammation of dental pulp, neuropeptides (inflammatory mediators) can induce the secretion of Leptin. Thus Leptin could be considered as an inflammatory mediator which is consistent with our findings.

## Conclusion 

Leptin can be considered as an inflammatory mediator during the early phases of dental periapical lesions and it may also have a role in repair as shown by other studies. Further studies are needed in order to clarify the precise role of Leptin.
